# Pancreatic Neuroendocrine Microtumors (WHO 2022) Are Not Always Low-Grade Neoplasms: A Case with a Highly Increased Proliferation Rate

**DOI:** 10.1007/s12022-024-09802-7

**Published:** 2024-02-26

**Authors:** Aziz Chouchane, Philipp Kirchner, Ilaria Marinoni, Eva Sticová, Tomáš Jirásek, Aurel Perren

**Affiliations:** 1 Institute For Tissue Medicine and Pathology, University of Bern, Bern, Switzerland; 2 Clinical and Transplant Pathology Centre, Institute of Clinical and Experimental Medicine, Prague, Czech Republic; 3 Department of Pathology, Liberec Regional Hospital, Liberec, Czech Republic

**Keywords:** PanNETs, Pancreatic neuroendocrine microtumors, Neuroendocrine neoplasm, Microadenoma, WHO 2022, Ki-67, MEN1

## Abstract

Traditionally considered non-functional low proliferative benign neuroendocrine proliferations measuring less than 5 mm, pancreatic (neuro)endocrine microadenomas are now classified as pancreatic neuroendocrine microtumors in the 2022 WHO classification of endocrine and neuroendocrine tumors. This case report discussed the features of an incidentally identified 4.7-mm glucagon-expressing pancreatic neuroendocrine microtumor with *MEN1* mutation only, chromosomally stable and an epigenetic alpha-like phenotype. The tumor was associated with an unexplained increased proliferation rate in Ki-67 of 15%. There was no associated DAXX/ATRX deficiency. The presented case challenges the conventional thought of a low proliferative disease of the so-called “pancreatic neuroendocrine microadenomas” and provides additional support to the 2022 WHO classification that also requires grading of these neoplasms. Despite exhibiting molecular features of less aggressive behavior, the case also underscores the biological complexity of pancreatic neuroendocrine microtumors. By recognizing the heterogenous spectrum of neuroendocrine neoplasms, the current case also contributes to ongoing discussions on how to optimize the clinical management of such tumors.

## Introduction

Non-functioning pancreatic neuroendocrine microtumor is a rare pathological entity generally associated with genetic syndromes including but not limited to MEN1/MEN4 syndromes and VHL disease. Nevertheless, there has been a steady rise in incidental findings as a result of more advanced screening methods. The 2022 WHO classification of endocrine and neuroendocrine tumors categorizes all pancreatic neuroendocrine microtumors as malignant [1]. However, it is well recognized that the majority of these tumors exhibit histological and biological features of low-grade malignancy and have a slow and non-aggressive clinical course. Various molecular and biological characteristics, such as mutations in the *DAXX/ATRX* genes, elevated proliferation rate, and the presence of alternative lengthening of telomeres (ALT), have been associated with a more rapid progression, a more aggressive disease course, and a higher likelihood of recurrence [2, 3]. Moreover, assessing the proliferation rate of these tumors by quantifying the mitotic index and measuring the Ki-67 index is an integral part in the histological grading of these neoplasms, differentiating between well-differentiated and poorly differentiated epithelial neuroendocrine neoplasms. In this case report, we investigated an incidentally identified unusually highly proliferative pancreatic neuroendocrine microtumor that exhibits a Ki-67 and mitotic index of a G2 disease but does not harbor any of the molecular features that are associated with the usual poorer outcomes. This case not only emphasizes the need for further research on the biology of pancreatic neuroendocrine tumors (PanNETs), including that of microtumors, but also raises questions about the treatment options for patients. The current European guidelines are increasingly favoring a less aggressive therapeutic approach focused on observation and control rather than intensive treatment [4, 5].

## Case Presentation

**Fig. 1:**
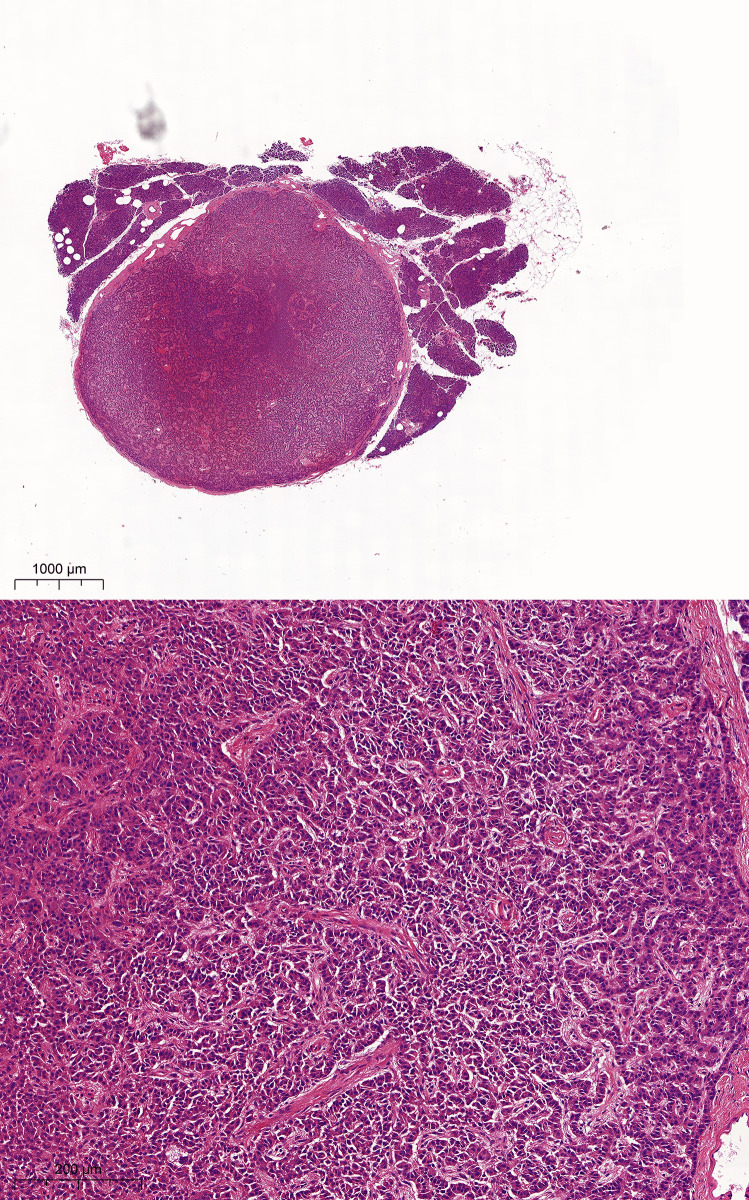
Histologic features of the pancreatic neuroendocrine microtumor. Routine H&E staining shows a well-demarcated, round mass in the middle of regular pancreatic parenchyma. High magnification image shows trabecular nests of small, round uniform cells with a low degree of atypia between fibrous septae

A 4.7-mm-diameter, well-delineated round mass was identified macroscopically during the perioperative examination of the pancreas of a 59-year-old female organ donor. This well-circumscribed lesion replaced the normal pancreatic parenchyma at the microscopic level. The lesion had the typical histomorphology characteristics of a neuroendocrine tumor, consisting of solid nests with small, round, and uniform cells with a salt and pepper chromatin (Fig. 1). The mitotic index was determined to be 2 mitoses per 2 mm^2^.

**Fig. 2:**
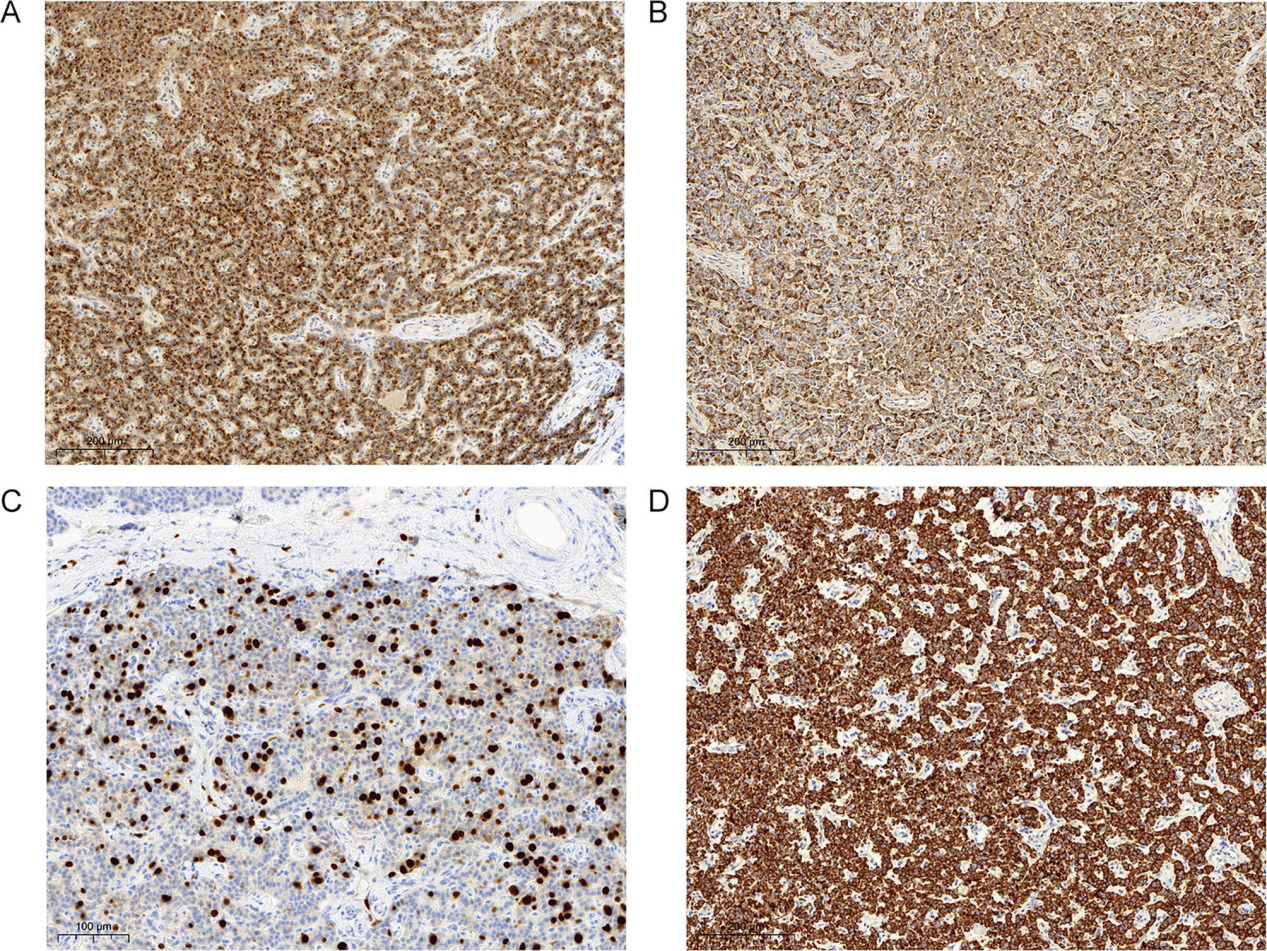
Immunohistochemical features. The neuroendocrine microtumor showed diffuse cytoplasmic positivity for chromogranin A (**A**) and glucagon (**B**). Ki-67 staining showed a proliferative “hotspot” of up to 15% (**C**), and CK8/18 (**D**) staining showed a strong membranous positivity

The morphological diagnosis was confirmed by immunohistochemical positivity for synaptophysin, chromogranin-A, and CK8/18 during the routine workup. Immunohistochemistry revealed glucagon monohormonal staining with no staining for insulin, somatostatin, serotonin, or pancreatic polypeptide (PP). Unexpectedly, the Ki-67 index was found in up to 15% of tumor cells in hotspots and was therefore substantially increased (Fig. 2). *DAXX* and *ATRX* wild-type expression was conserved with a strong nuclear staining. The neuroendocrine microtumor shows no reactivity to carbonic anhydrase 9 (CA9) and alpha-inhibin.

Targeted Next Generation Sequencing (NGS) on the tumor DNA, using the TSO500 assay, unveiled a missense mutation c.841G > A in the *MEN1* gene. This rare variant results in the substitution of glycine by arginine in the *MEN1* protein, i.e., p.(Gly281Arg).

**Fig. 3:**
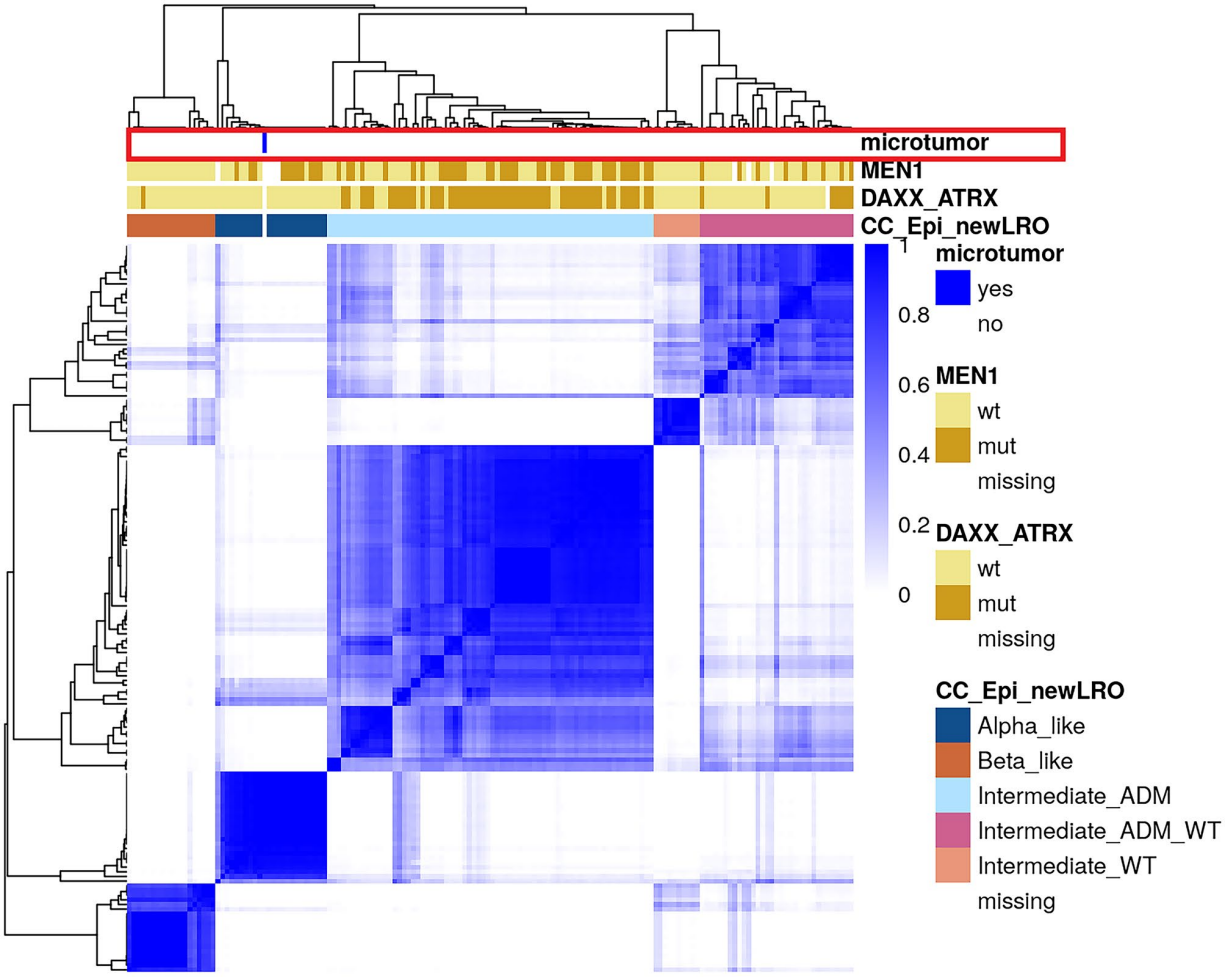
Hierarchical Cluster Analysis of the pancreatic neuroendocrine tumor based on methylation profiling. The methylation profile of the pancreatic neuroendocrine microtumor when compared to the tumors analyzed by De Domenico et al. shows a similar epigenetic profile to the “*MEN1* mutation only” subgroup [2]

Whole genome methylation analysis using EPIC arrays showed an alpha-like epigenetic pattern with high similarity to alpha-cells. This group corresponds to the “*MEN1* mutation only” genotype, which was associated with a lack of relapse in a retrospective analysis [2] (Fig. 3). No copy number changes were observed.

In summary, we present a glucagon-expressing pancreatic neuroendocrine microtumor with *MEN1* mutation only, chromosomally stable with an epigenetic alpha-like phenotype, unexplained increased proliferation rate in Ki-67 of 15%.

## Discussion

In 2017, the WHO defined Non-Functioning Pancreatic Neuroendocrine Tumors (NF-PanNETs) smaller than 5 mm as microadenomas and considered them benign [1]. In their updated 2022 definition, NF-PanNETs started to be considered malignant regardless of their size, due to a report of lymph node metastases in a case of NF-PanNETs smaller than 5 mm [6] as well as WHO expert observations on the spectrum of their proliferative activity, similar to the current case [1]. The pancreatic neuroendocrine microtumor in that case measured 0.4 cm and showed insulin reactivity along with a Ki67 labeling index of 0.85%.

Pancreatic neuroendocrine microtumors are generally considered precursors to larger PanNETs, especially in the context of hereditary disease. Neuroendocrine microtumors frequently have Ki-67 index values below 1% due to their largely non-metastatic nature [1]. In the current case report, we described a patient who had a pancreatic neuroendocrine microtumor that was less than 5 mm (4.7 mm) in size but showed an unusually high Ki-67 proliferation index. The neuroendocrine microtumor featured “proliferative hotspots” where the Ki-67 index reached a maximum of 15% positivity. These features are quite different than the pancreatic neuroendocrine microtumor described in the case that prompted the nomenclature change. Is this an indication that this microtumor has a higher potential for disease progress?

In analogy to mouse models, Sadanandam et al. [7] have simultaneously proposed the concepts of “metastasis-like primary tumors” and “well-differentiated/islet” tumors in PanNETs, describing very small lesions (microtumors) with contrasting RNA signatures. These signatures were defined by specific epigenetic, metabolic, and developmental properties in both rodent and human models. In general, the metastasis-like primary is characterized by hypoxia and stem cell gene signatures, whereas the well-differentiated subtype relates to genes expressed in insulinomas (*INSM1*, *INS*). Because of this variation in gene expression, metastasis-like tumors are more comparable to immature β cells than to well-differentiated tumors, which in turn resemble adult islet β cells on a molecular level. As previously stated, the pancreatic neuroendocrine microtumor in this current case has a molecular profile more similar to alpha than to beta cells, with a *MEN1* mutation and wild-type *DAXX*/*ATRX* expression, as well as no evidence of hypoxia or pseudohypoxia with no immunohistochemical reactivity to CA9. CA9 is a transmembrane protein that has been shown to be overexpressed in a wide variety of epithelial and non-epithelial neoplasms particularly in the context of (pseudo) hypoxia-driven pathogenesis [8, 9]. More specifically, in sporadic neuroendocrine tumors of the pancreas, overexpression of CA9 was shown to be strongly correlated to modifications of the *VHL* gene with subsequent activation of the *HIF1*-alpha pathway. These PanNETs with CA9 overexpression have been shown to follow a more aggressive disease course [10, 11]. The described characteristics above argue against the classification of this pancreatic neuroendocrine microtumor as a “metastasis-like primary.” Interestingly, the metastasizing pancreatic neuroendocrine microtumor described by Kwon et al. [6] showed monohormonal positivity for insulin, making the case that it could belong to the “metastasis-like” primary subgroup.

Although *DAXX* and *ATRX* mutations are associated with a more aggressive disease course in PanNETs [2, 3, 12], the pancreatic neuroendocrine microtumor in our current case retained wildtype expression of both *DAXX* and *ATRX* while exhibiting an aggressive feature with a high proliferation rate. This discrepancy confirms the degree of heterogeneity in NF-PanNETs and raises further concerns regarding the clinical management of patients with comparable results. To determine the optimal management strategy for asymptomatic sporadic PanNETs less than 2 cm, multinational partners in the field of PanNETs made the decision to launch the ASPEN (Asymptomatic Sporadic Nonfunctioning Pancreatic Neuroendocrine Neoplasms) project [5, 13]. The ASPEN trial’s objectives are to undertake prospective research and develop solid guidelines for the treatment and management of small NF-PanNETs, a topic of ongoing discussion among experts.

Based on the preliminary findings, the authors of an interim analysis of the ASPEN clinical trials draw the conclusion that a non-operative approach does not appear to be inferior to surgical resection due to the fact that only a small percentage of patients experienced an increase in tumor size and none of them experienced distant metastases [5]. The patient’s desires and the young age of the patient were the main factors in cases where surgical resection of the pancreatic neuroendocrine microtumor was recommended.

The European Neuroendocrine Tumor Society currently recommends a wait-and-observe protocol [4], which is in line with ASPEN’s preliminary findings.

In summary, we described a very unusual non-functioning pancreatic neuroendocrine microtumor that was discovered incidentally during the pretransplant evaluation of the organ donor, demonstrating an unusually high rate of proliferation, but exhibits metabolic, genetic, and epigenetic features that are more correlated with a less aggressive disease course. Despite very thorough molecular analyses, we were unable to find an explanation for this increase in proliferation rate, underlying that we still lack an important understanding of PanNET tumorigenesis, even at the state of microtumors (< 5 mm). More recently, specific histomorphological features such as tumor vascular invasion or the evidence of an infiltrative growth pattern have been shown to be quite reliable predictors of distant metastasis and adverse outcomes [14–17], but we hypothesize that with the singular increase of Ki-67, without any other aggressive molecular features or the above mentioned morphological signs, the risk of metastases in the patient that received a liver transplant from the autopsied individual is low. Despite the low risk of progression, a follow-up sonography was performed a year after the transplantation to exclude the emergence of any adverse events. The sonography showed a slightly enlarged reduced-size liver graft with no signs of pathological or suspect changes.

## Data Availability

Methylation data available on request via the corresponding author.
